# COVID-19 detection using federated machine learning

**DOI:** 10.1371/journal.pone.0252573

**Published:** 2021-06-08

**Authors:** Mustafa Abdul Salam, Sanaa Taha, Mohamed Ramadan

**Affiliations:** 1 Artificial Intelligence Department, Faculty of Computers and Artificial intelligence, Benha University, Benha, Egypt; 2 Information Technology Department, Faculty of Computers and Artificial Intelligence, Cairo University, Giza, Egypt; 3 Computer Science Department, Faculty of Computers and Information- Egyptian E-Learning University, Giza, Egypt; Vellore Institute of Technology: VIT University, INDIA

## Abstract

The current COVID-19 pandemic threatens human life, health, and productivity. AI plays an essential role in COVID-19 case classification as we can apply machine learning models on COVID-19 case data to predict infectious cases and recovery rates using chest x-ray. Accessing patient’s private data violates patient privacy and traditional machine learning model requires accessing or transferring whole data to train the model. In recent years, there has been increasing interest in federated machine learning, as it provides an effective solution for data privacy, centralized computation, and high computation power. In this paper, we studied the efficacy of federated learning versus traditional learning by developing two machine learning models (a federated learning model and a traditional machine learning model)using Keras and TensorFlow federated, we used a descriptive dataset and chest x-ray (CXR) images from COVID-19 patients. During the model training stage, we tried to identify which factors affect model prediction accuracy and loss like activation function, model optimizer, learning rate, number of rounds, and data Size, we kept recording and plotting the model loss and prediction accuracy per each training round, to identify which factors affect the model performance, and we found that softmax activation function and SGD optimizer give better prediction accuracy and loss, changing the number of rounds and learning rate has slightly effect on model prediction accuracy and prediction loss but increasing the data size did not have any effect on model prediction accuracy and prediction loss. finally, we build a comparison between the proposed models’ loss, accuracy, and performance speed, the results demonstrate that the federated machine learning model has a better prediction accuracy and loss but higher performance time than the traditional machine learning model.

## Introduction

### COVID-19

The current COVID-19 pandemic, caused by SARS CoV2, threatens human life, health, and productivity [1] and is rapidly spreading worldwide [2]. The COVID-19 virus, like other family members, is sensitive to ultraviolet rays and heat [3]. AI and deep learning play an essential role in COVID-19 cases identification and classification using computer-aided applications, which achieves excellent results for identifying COVID-19 cases [1] based on known symptoms including fever, chills, dry cough, and a positive x-rays. AI, and the deep learning model can be used to forecast the spread of the virus based on historical data which can help control its spread [3]. So there is a need to build machine learning models to identify COVID-19 infected patient or to predict the spread of the virus in the future, but this is not easy to achieve because patient data is confidential, and without enough data, it is too difficult to build a robust model [1]. A new approach is needed that makes it easy to build a model without accessing a patient’s private data or requires transferring patient’s raw data, and one which gives high prediction accuracy.

### Federated learning

The concept of federated learning was proposed by Google in 2016 as a new machine learning paradigm. The objective of federated learning is to build a machine learning model based on distributed datasets without sharing raw data while preserving data privacy [4, 5].

**Fig 1:**
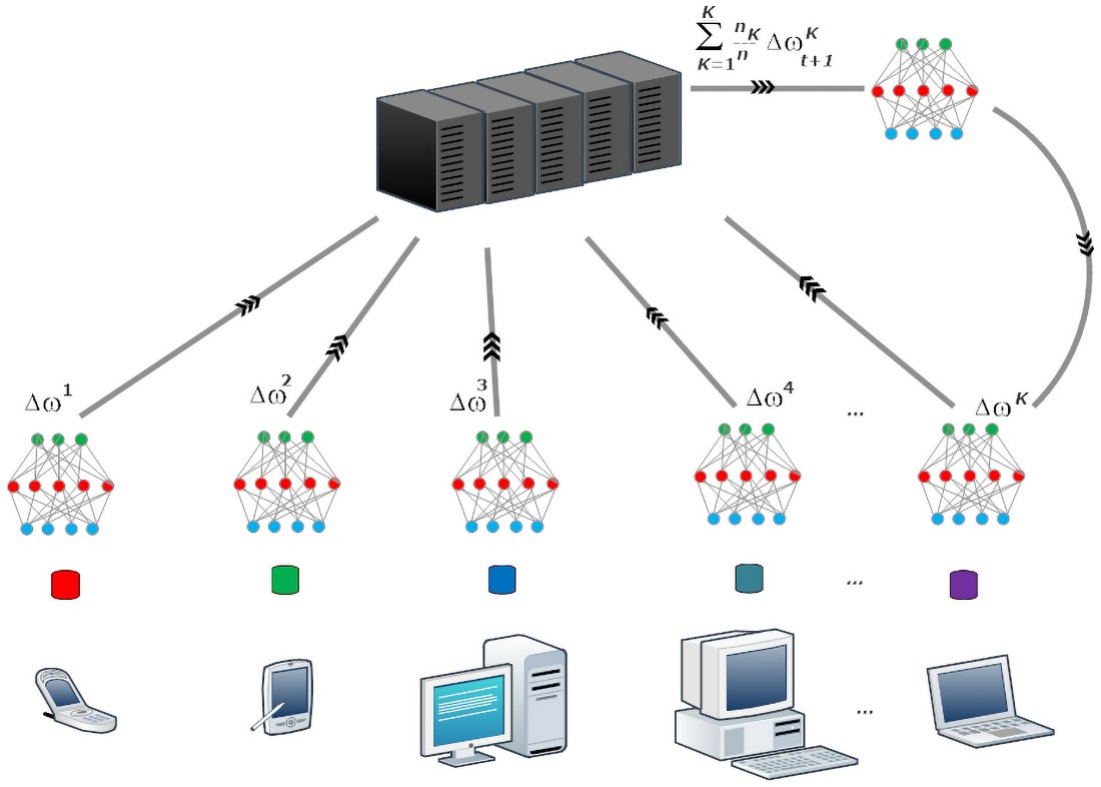
The global model collects local models updates.

In federated machine learning, each client (organization, server, mobile device, and IoT device) has a dataset and his local machine learning model. There is a centralized global server in a federated environment that has a centralized machine learning model (global model), which aggregates the distributed client’s model parameters (model gradients). Each client trains the local machine learning model locally on a dataset and shares the model parameters or wights to the global model. The global model makes iteration of rounds to collect the distributed clients model updates without sharing raw data [4, 5] as shown in Fig 1.

Why federated machine learning should be used:

- Decentralized model removes the need to transfer all the data to one server to train the model, as training each node occurs locally, unlike traditional machine learning which requires moving all the data to a centralized server, to build and train the model.
- No data privacy violation as it applies methodologies including the differential privacy and the homographic Secure multiparty computation, unlike traditional machine learning.
- A third-party can be part of the training process as long as there is no data privacy violation and data is secured, unlike traditional machine learning third-party could not be an option in case of military organizations.
- Less computation power is needed as model training is performed on each client, and the centralized model’s primary role is to collect gradient update distributed models, unlike the traditional machine learning which one centralized server contains all the data, which requires high computational power for model training.
- Decentralized algorithms may provide better or the same performance as centralized algorithms [5].

It is highly recommended to use federated machine learning rather than traditional machine learning, in such environments where data privacy, is highly required. Federated learning can be applied in many disciplines like (Smart healthcare, sales, multi-party database, and smart retail) [6]

## Motivation and contributions

Federated machine learning enables us to overcome the obstacles faced by the traditional machine learning model as:

- Traditional machine learning occurs by moving all data source to a centralized server to train and build the model, but this may violate the rules of military organizations especially when third-party is used to create, train and maintain the model.
- To train the model, the third-party should prepare, clean, and restructure the data to be suitable for model training, however, this may violate data privacy when the data are handled to create the model.
- Traditional machine learning models also take much time to build the model with acceptable accuracy, which may cause a delay for organizations, especially recently opened ones.
- Traditional machine learning also requires the existence of a massive amount of historical data to train the model to give acceptable accuracy (Cold Start) [7].
- There is a need for a secure distributed machine learning methodology that trains clients’ data on their servers without violating data privacy, saves computational power, and overcomes the cold start problem, enabling clients to get immediate results.

Federated learning has the potential to solve these issues, as it enables soiled data servers to train their models locally and to share their model’s gradients without violating patient privacy [1].

The principal objective of this paper is, to build a comparison between a federated machine learning model and a non-federated machine learning model, by applying them to the same datasets and build the comparison between the model’s prediction loss, prediction accuracy, and training time.

## Related work

*Boyi Liu et al.* [1] proposed an experiment to compare the performance of federated machine learning, between four popular models(Mobile Net, ResNet18, MobileNet-v2, and COVID-Net), by applying them to the patient’s chest images CXR dataset. These models are designed to recognize COVID-19 pneumonia, the authors used the same parameters for all models, after 100 rounds the authors found that the ResNet18 model is the fastest model and gives the highest accuracy rate (96.15%, 91.26%), second, the COVID-Net and MobileNet-v2 had the same loss value as COVID-Net and Mobile Net. Non-federated learning was conducted on the same data and it was found that the loss convergence rate caused by using federated learning decreased slightly.

*Junjie Pang et al.* [8] proposed a federated learning framework based on digital city twin concepts to study the effect of different prevention city plans to prevent a COVID-19 outbreak, and by building a federated model to predict the effect they traced the infection number from multiple cities over the periods from their digital city twin systems. They were also able to trace the effectiveness of each prevention plan and build local models on each digital city twin system which sent the model parameters or updates to federated sites to maintain data privacy. They built a comparison between the prediction accuracy and loss between the federated model and the traditional one.

*Weishan Zhang et al.* [4] proposed a novel dynamic fusion-based federated learning approach to enhance federated learning model performance metrics. They found that all the recent studies on federated learning used the default federated learning settings which may introduce huge communication overhead and underperforms when there is data heterogeneity between clients. They proposed an approach which determines the interaction between clients and servers with a dynamic fusion-based function to determine which client participates in each round to upload his local model updates.

They defined a max waiting time for each client to participate during the server round which was defined by the platform owner. They applied four models using this architecture. GhostNet, ResNet50, and ResNet101 were used on COVID-19 datasets and they found that the proposed approach introduce better accuracy than the default setting one and can reduce communication overhead and the training time for ResNet50 and ResNet101, however, these results did not apply to GhostNet.

*Parnian Afshar et al.* [9] proposed a modeling framework based on capsule networks (COVID-CAPS) to identify positive COVID-19 cases from x-rays images to overcome the drawbacks of CNN-based models for handling small dataset, they modified the model parameters to perform well and conducted a comparison between the COVID-CAPS and the traditional network and found that the COVID-CAPS model performed better than the traditional model for accuracy, sensitivity, and specificity

*Chaoyang He et al.* [10] proposed an experimental study on automating federated learning (AutoFL) using the Neural Architecture Search (NAS) algorithm and proposed a Federated NAS (FedNAS) algorithm to find the optimal design settings of local machine learning models to obtain the performance and effectiveness of the local models that share their model updates. They found that default settings of local machine learning models did not fit the federated environment nature as the clients contain non-identical and non-independent data i.e., non-IID clients. The experiment was conducted using the CIFAR10 dataset and found that FedNAS can search for a better architecture with an 81.24%accuracy in only a few hours compared to 77.78% for FedAvg.

*Amir Ahmad et al.* [11] proposed a detailed literature review of start-of-art taxonomies used in COVID-19 case prediction and they categorized them into four categories. The authors built a comprehensive review to provide suggestions to machine learning practitioners to improve the accuracy of their machine learning model and the challenges that they may face.

*Nikos Tsiknakis et al.* [12] introduced a study on COVID-19 classification using the transfer learning method which achieves better AUC performance; their study proposed a deep learning-based COVID-19 classification system based on x-rays for better performance compared to state–of–the–art methodologies.

*Mwaffaq Otoom et al.* [13] proposed a real-time COVID-19 case detection and monitoring system. Their study used an IOT device for data collection and monitoring during quarantine; they used seven machine learning algorithms and conduct an experiment on each experiment and build a comparison they found that five machine learning algorithms had greater than 90% prediction accuracy.

*Thanh Thi Nguyen et al.* [14] proposed a survey of AI methods used in various applications used in fighting COVID-19; they covered areas including data analytics, data mining, and natural language processing (NLP). The authors identified previous problems and identified the solutions based on COVID-19 AI methods on chest x-rays image datasets.

*Fatima M Salman et al.* [15] Proposed Machine learning model to identify COVID-19 cases using patient’s chest x-rays images by implementing convolutional neural network CNN machine learning algorithm, they used patient’s chest x-rays datasets contains 130 images of COVID-19 x-ray cases and 130 images for normal cases x-ray, their prediction machine learning model gives 100% prediction accuracy.

*N Narayan Das et al.* [16] proposed a machine learning model to identify COVID-19 cases using patient’s chest x-rays images by implementing the Inception (Xception) machine learning model. They overcame the RT-PCR kits issues due to the time and cost required to identify the COVID-19 cases by using patient’s chest x-rays images; their models outperform competitive models.

*AKMB Haque et al.* [17] proposed a study on how to detect Covid-19, pneumonia, and normal chest cases using patient’s chest x-rays images by implementing the different Convolutional Pre-Trained Neural Network models (VGG16, VGG19, Xception, InceptionV3, and Resnet50). They found that VGG16 and VGG19 showed high performance and prediction accuracy, and also investigated the effects of weather factors including temperature, humidity, sun hour, and wind speed and found that temperature had a great effect on death cases caused by Covid-19.

*Himadri Mukherjee et al.* [18] proposed a machine learning model to identify COVID-19 cases using patient’s chest CT scan or CXR images by implementing the Convolutional Neural Network (CNN)-tailored Deep Neural Network (DNN) machine learning algorithm, they found that the proposed model achieves overall high accuracy compared with others models like InceptionV3, MobileNet, and ResNet.

*Ike FIBRIANI et al.* [19] proposed a machine learning model to identify COVID-19 cases using patient’s chest X-ray images by implementing a multi-layer Convolutional Neural Network (CNN) machine learning algorithm; they created a multi–Convolutional Neural Network (CNN) classifier architecture to minimize the errors and found that the majority vote and the proposed model achieves high accuracy.

*Harsh Panwar et al.* [20] proposed a machine learning model to identify COVID-19 cases using patient’s chest X-ray images by implementing a deep learning neural network-based method nCOVnet, and found that the machine learning model gives high prediction accuracy.

*Shashank Vaid, et al.* [21] proposed a machine learning model to uncover the hidden patterns that exist between COVID-19 cases to predict the potential infection. They used their model to identify the key parameter that used to detect the hidden patterns between cases (dimensionality reduction) then applied their model using the unbiased hierarchical Bayesian estimator.

*Rodrigo M Carrillo-Larco et al.* [22] proposed a machine learning model to group countries with shared COVID-19 infection profiles. They used unsupervised machine learning algorithms (k-means), and collect data from COVID-19 cases from 155 countries, and implemented the K-mean clustering algorithm and principal component analysis (PCA) to group the countries.

*Fadoua Khmaissia et al.* [23] proposed an unsupervised machine learning model to find similarities between zip codes in New York City to study COVID-19 inside the city. They used feature selection and clustering techniques to find similarities based on mobility, socioeconomic, and demographic features with the COVID-19 trends.

*Akib Mohi Ud Din Khanday et al.* [24] proposed an unsupervised machine learning model to classify textual clinical reports in four classes to study the Behavior of COVID-19. They used Term frequency/inverse document frequency (TF/IDF), bag of words (BOW), and report length to generate features and used these features for traditional machine learning algorithms to generate better results and found that it gives better testing accuracy.

*R Manavalan et al.* [25] proposed a study to explore the association between COVID-19 transmission rates and meteorological parameters by implementing a gradient boosting model (GBM) on Indian data. GBM model was optimized after tuning its parameters.

*Sina F Ardabili et al.* [26] proposed a study to compare between machine learning and soft computing models in predict the COVID-19 outbreak and built a comparative analysis, which found that multi-layered perceptron (MLP), and adaptive network-based fuzzy inference system, (ANFIS) shows a promise.

*Sara Hosseinzadeh Kassan et al.* [27] proposed a study comparing between most popular deep learning-based feature extractions frameworks like MobileNet, DenseNet, Xception, ResNet, InceptionV3, InceptionResNetV2, VGGNet, and NASNet by applying to COVID-19 chest X-rays patients to help in COVID-19 automatic detection. They found that DenseNet121 feature extractor with Bagging tree classifier achieved the best performance.

*Iwendi, Celestine, et al.* [28] proposed a system for classifying and analyzing the predictions obtained from COVID-19 symptoms, by using the Adaptive Neuro-Fuzzy Inference System (ANFIS), which helps in detecting Coronavirus Disease early. The authors found that the support vector machine (SVM) algorithm gives better prediction accuracy among all classifiers.

*Javed, Abdul Rehman, et al.* [29] presented a generalized collaborative framework named collaborative shared healthcare plan (CSHCP) used for people cognitive health and fitness assessment, the proposed framework shows promising outcomes compared to the existing studies.

*Bhattacharya, Sweta, et al.* [30] presented summarizing for start-of-art research works related to COVID-19 medical image processing deep learning applications, and provided an overview for deep learning applications used in healthcare in the last decade. Finally, they discussed the deep learning application’s challenges used in COVID-19 medical image processing.

*Manoj, Mk, et al.* [31] proposed incentive-based approach is provided to channel isolation which helps the people in need during these tough times and proposed also a blockchain-based solution to prevent information tampering.

*Reddy, G. Thippa, et al.* [32] proposed an experiment using an adaptive genetic algorithm with fuzzy logic (AGAFL) model to predict heart disease which helps practitioners to early diagnosing heart disease, they applied the proposed model on UCI heart disease dataset and found that the proposed approach is outperformed current methods.

*Anwaar Ulhaq et al.* [33] introduced a theoretical framework called differential privacy by design (dPbD) that helps to design scalable and robust federated machine learning systems for COVID-19 data privacy. Privacy by design embeds privacy directly into the system design and was introduced by [34], authors found that all studies focused on the tradeoff between privacy and utility and ignored the system scalability (number of clients attached) and robustness (the performance of the system against attacks) so they define seven steps as a theoretical framework to be applied when using federated machine learning.

## Materials and methods

This section addresses the applied tools and methodology for the federated and traditional one, to predict recovery based on the features of the patient. Tensor flow with Keras API was used to build federated and traditional mode, following steps were used for building models:

### The federated learning model

Algorithm 1. The Federated Learning Model

Input: COVID-19 Dataset as CSV file

Output: Model Prediction Accuracy and loss

Initialization:

- Data Loading (data loaded using pandas package which returned data frame object with data).
- Drop Unique Values Column (all unique, primary keys, and distinct values columns had dropped during model training).
- Replace Null Values (the null values were replaced with mode values to make it easy to model for data training).
- Label Encoding (categorical label and text labels were replaced with normalized values).
- Data Repetition (data were repeated to simulate the number of clients).
- Data Shuffling (data shuffled to avoid getting the same results).
- Data Batching (data grouped into batches to enhance performance).
- Data Mapping (ndarray dataset flattened to 1 darray dataset).
- Data Prefetching (data cached in memory for better performance).
- Create Deep Learning Model (sequential deep learning model built using Keras API).
- Create Federated Learning Model (using Keras API from_keras_model deep learning model wrapped and built a federated learning model).
- Create a Federated Average Process (collecting local models gradients and updates to be sent to the global model).
- Model Initializing and Training (iterative process initialized and start training).
- Model Evaluation (the model performance was evaluated by print evaluation metrics).
- Return the machine learning model accuracy and loss for each round.

### The traditional machine learning model

Algorithm 2. The Traditional Machine Learning Model

Input: COVID-19 Dataset as CSV file

Output: Model Prediction Accuracy and loss

Initialization:

- Data Loading (data loaded by pandas package which returned data frame object with data).
- Drop Unique Values Column (all unique, primary keys, and distinct values columns had dropped during model training).
- Replace Null Values (the null values replaced with mode values to make it easy to model for data training).
- Label Encoding (categorical label and text labels replaced with normalized values).
- Create Deep Learning Model (sequential deep learning model built using Keras API).
- Model Evaluation (the model performance was evaluated by print evaluation metrics).
- Return the machine learning model accuracy and loss for each round.

## The proposed model

### Federated learning model on patient’s chest x-rays images

**Fig 2:**
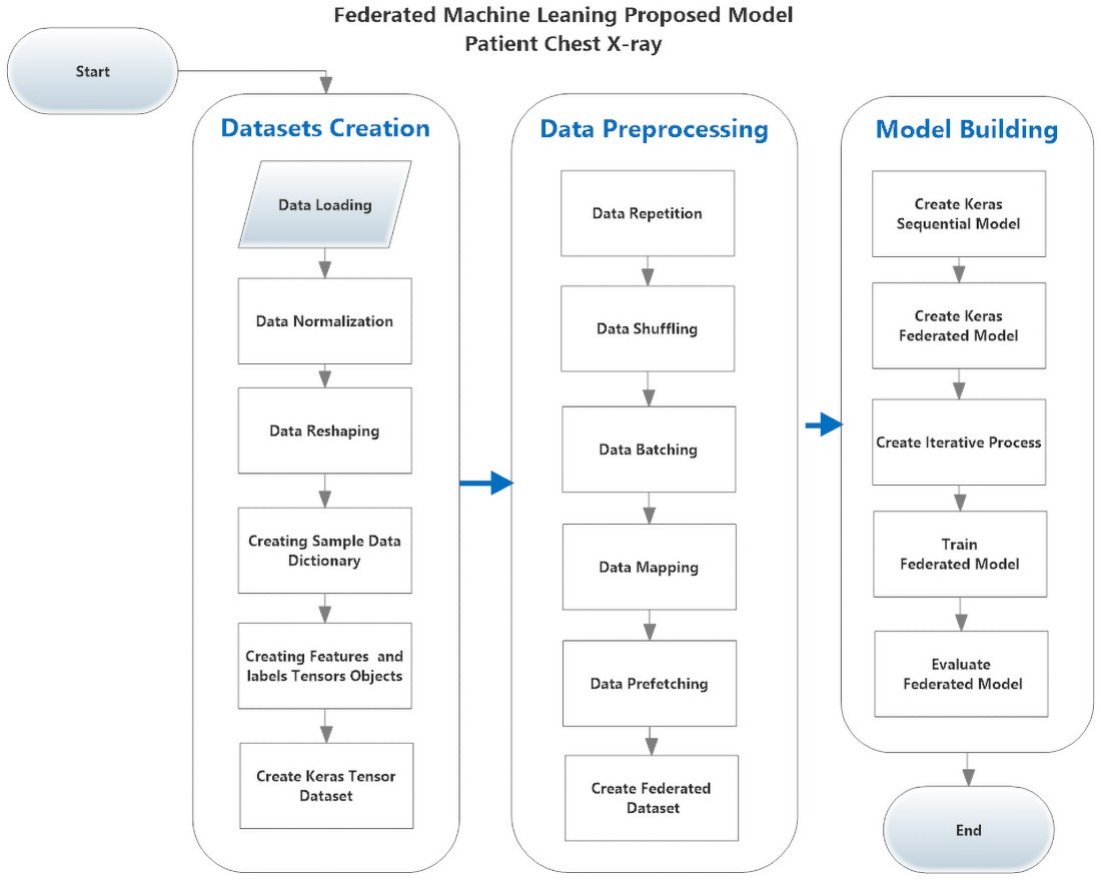
The proposed federated model for classifying COVID-19 cases from patient’s chest x-ray images.

As shown in Fig 2, the proposed federated model building steps are:

- Data Loading
  CV2 package was used to read chest x-ray images from the dataset download directory, and was loaded it into the memory object. The images were resized to 244*244*3 as color images.
- Data Normalizing
  Image data was divided by 255 to normalize it between 1 and 0.
- Data Reshaping
  The image object is an array of (244, 244, 3) should be flattened to be list (178, 608).
- Creating Sample Data Dictionary
  After flattening the data dictionary instance was created for each image sample to represent the image data (features) and its label.
- Creating Samples and labels Tensors keras Objects
  To build keras the dataset, the keras tensor object should be built for features and keras tensor object for labels.
- Create Keras Tensor Dataset
  Create keras dataset by using from_tensor_slices API.
- Data Repetition
  Data repeated to simulate the number of clients.
- Data Shuffling
  Data shuffled to avoid obtaining the same results.
- Data Batching
  Data grouped into batches to enhance their performance.
- Data Mapping
  ndarray dataset flattend to 1 darray dataset.
- Data Prefetching
  Data cached in memory for better performance.
- Create Keras Deep Learning Model
  Sequential deep learning model built using Keras API.
- Create Federated Learning Model
  Using Keras API from_keras_model deep learning model.
- Create a Federated Average Process
  collecting local models gradients and updates to be sent to the global model.
- Model Initializing and Training
  Initiated the iterative process and start training.
- Model Evaluation
  Evaluate the model performance by print evaluation metrics.

#### Federated model on patient’s descriptive data

**Fig 3:**
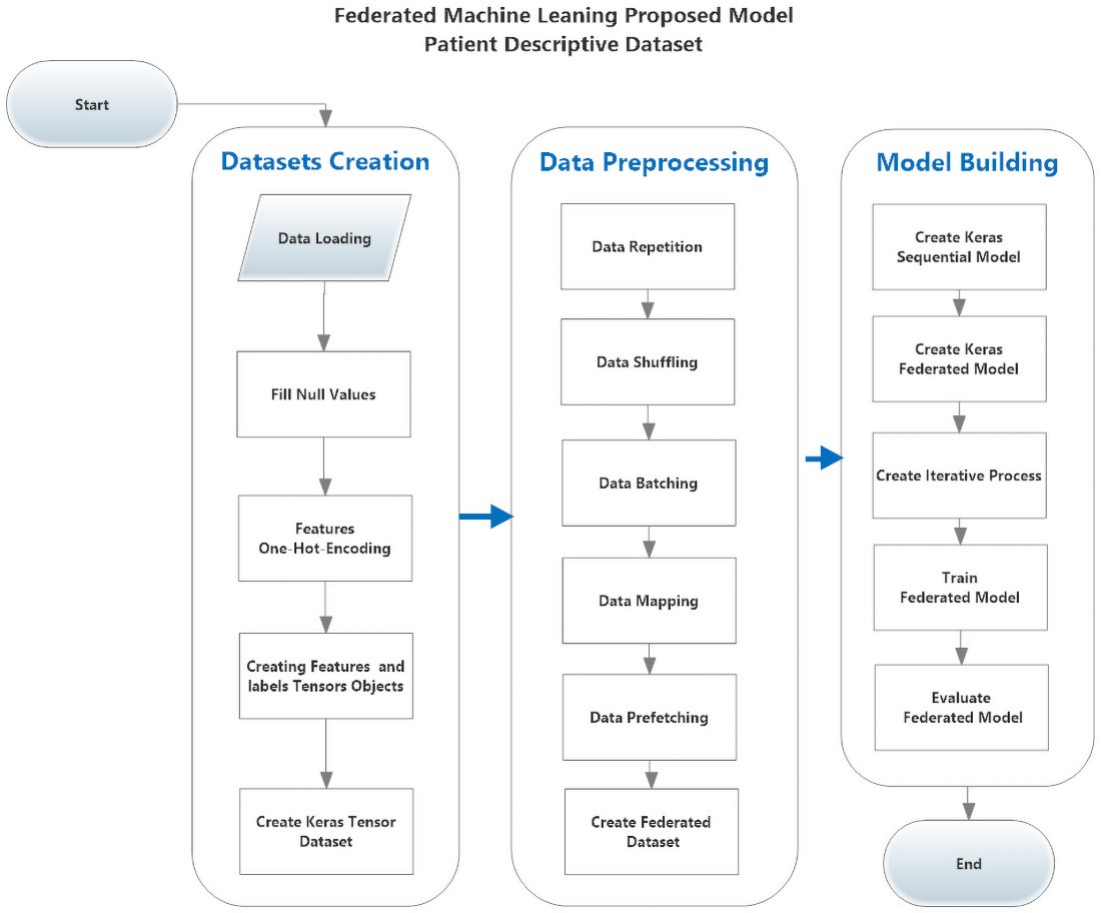
The proposed federated model for classifying COVID-19 cases from patient’s descriptive data.

As shown in Fig 3, to apply the same model to the patient’s descriptive dataset, there are modifications required to be done first, there no need for data normalization because the data is not all the same. There is no need for data reshaping step as the data is already flat, so there is a need to modify our model by removing some steps and adding new steps All of the data features are categorical so there is a need to encode it for processing, so there is a need to add a new step after creating the dataset for transforming categorical features to binary vectors, so model modifications can be summarized as follows:

Steps to be removed:

- Data Normalization.
  The data is not all the same type.
- Data Reshaping

Steps to be added

Features One-Hot Encoding.
  Convert features categorical values to binary vectors.

We modified the proposed model before model training with the Patient’s Descriptive Data.

#### Traditional model on patient’s chest x-ray images

**Fig 4:**
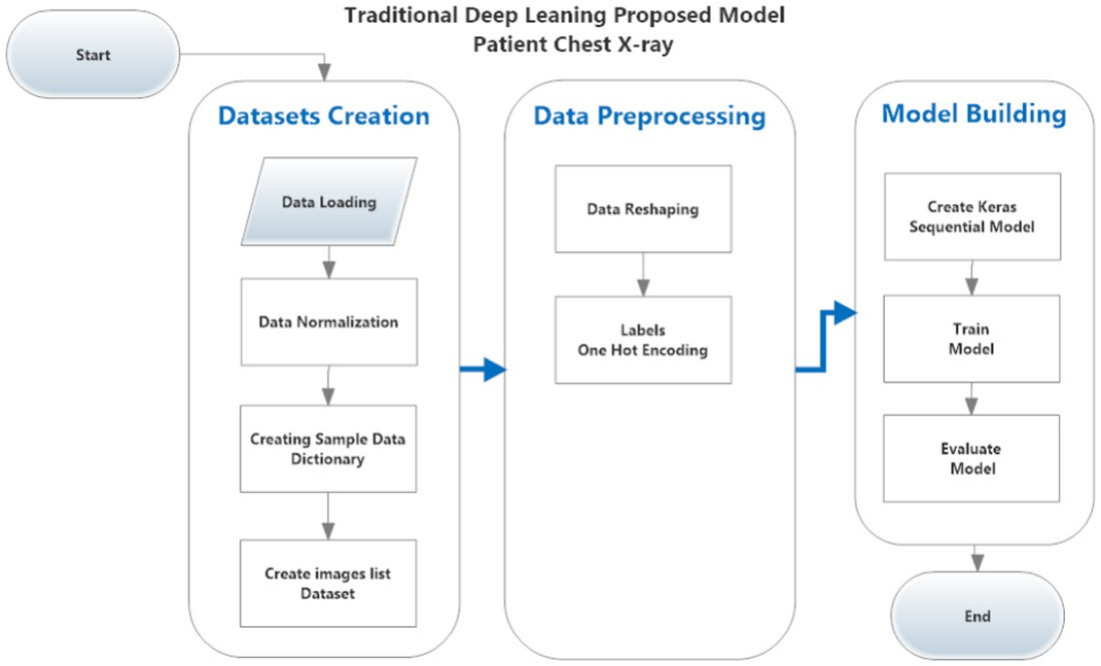
The proposed traditional model for classifying COVID-19 cases from chest x-ray images.

As shown in Fig 4 the proposed Traditional model building steps were:

- Data Loading
  CV2 package was used to read chest x-ray images from the dataset download directory, it was loaded it into the memory object. The images were resized to 244*244*3 as color images.
- Data Normalizing.
  Image data was divided by 255 to normalize it between 1 and 0.
- Creating Sample Data Dictionary.
  A dictionary instance was created for each image sample to represent the image data (features) and its label.
- Creating Samples and labels list Objects.
  List object were be built for features and labels.
- Data Reshaping.
  The image object was an array of (244, 244, 3) should be flattened to be listed(178, 608).
- Labels Encoding (categorical).
  To build a matrix of vectors of binary values representing categorical values of labels.
- Create Keras Deep Learning Model
  Sequential deep learning model created using Keras API.
- Model Initializing and Training.
  The iterative process initialized and start training.
- Model Evaluation.
  Model performance was evaluated by print evaluation metrics.

#### Traditional model on patient’s descriptive data

**Fig 5:**
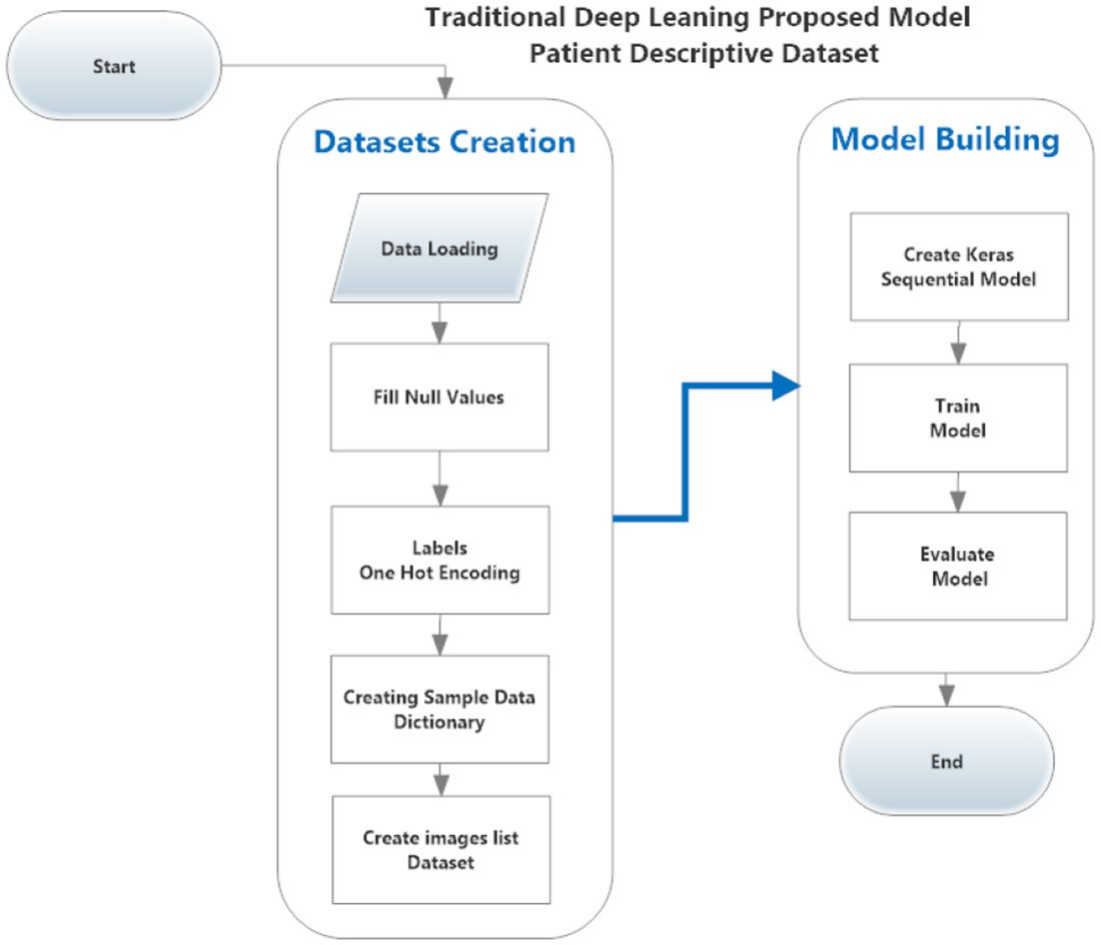
The proposed traditional model for classifying COVID-19 cases from patient’s descriptive dataset.

As shown in Fig 5, to apply the same model to the patient’s descriptive dataset, there are modifications required to be done first.

Steps to be removed:

- Data Normalization
- Data Reshaping

Steps to be added:

- features One-Hot Encoding(convert features categorical values to binary vectors).

## Results

### Patient’s descriptive COVID-19 datasets

**Fig 6:**
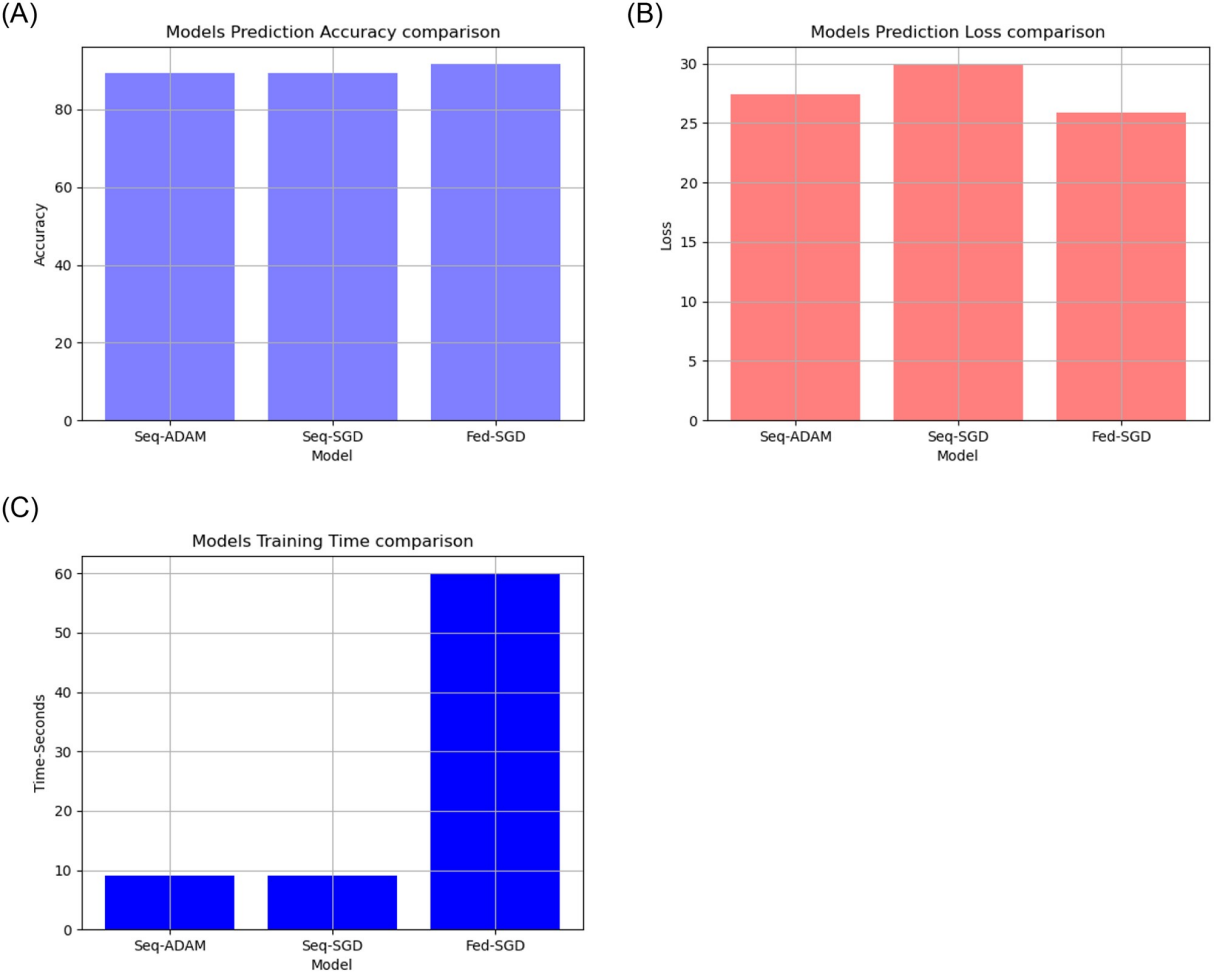
Model accuracy, loss and time comparison on descriptive patient’s dataset.

The patient’s descriptive COVID-19 datasets contained COVID-19 case information, and after training the two proposed models were used to predict the patient recovery rate. We found that:

- The proposed federated model had a higher prediction accuracy than the proposed traditional model As shown in Fig 6 and Table 1.
- The proposed federated model had lower prediction loss than the proposed traditional model As shown in Fig 6 and Table 1.
- The proposed federated model had high training time than the proposed traditional model As shown in Fig 6 and Table 1.

**Table 1:** Comparison between proposed models accuracy and loss on patient’s descriptive COVID-19 datasets.

Criteria	Sequential Model(ADAM)	Sequential Model(SGD)	Federated Model(SGD)
Optimizer	ADAM	SGD	SGD (learning_rate = 0.0001)
Loss Function	binary_crossentropy	binary_crossentropy	SparseCategoricalCrossentropy
Accuracy Function	accuracy	accuracy	SparseCategoricalAccuracy
Model Loss	27.38%	29.95%	25.87%
Model Accuracy	89.31%	89.31%	91.61%
Training Time	9 Seconds	9 Seconds	60 Seconds

### In patient’s chest x-ray radiography (CXR) images datasets

#### Binary classifier

**Fig 7:**
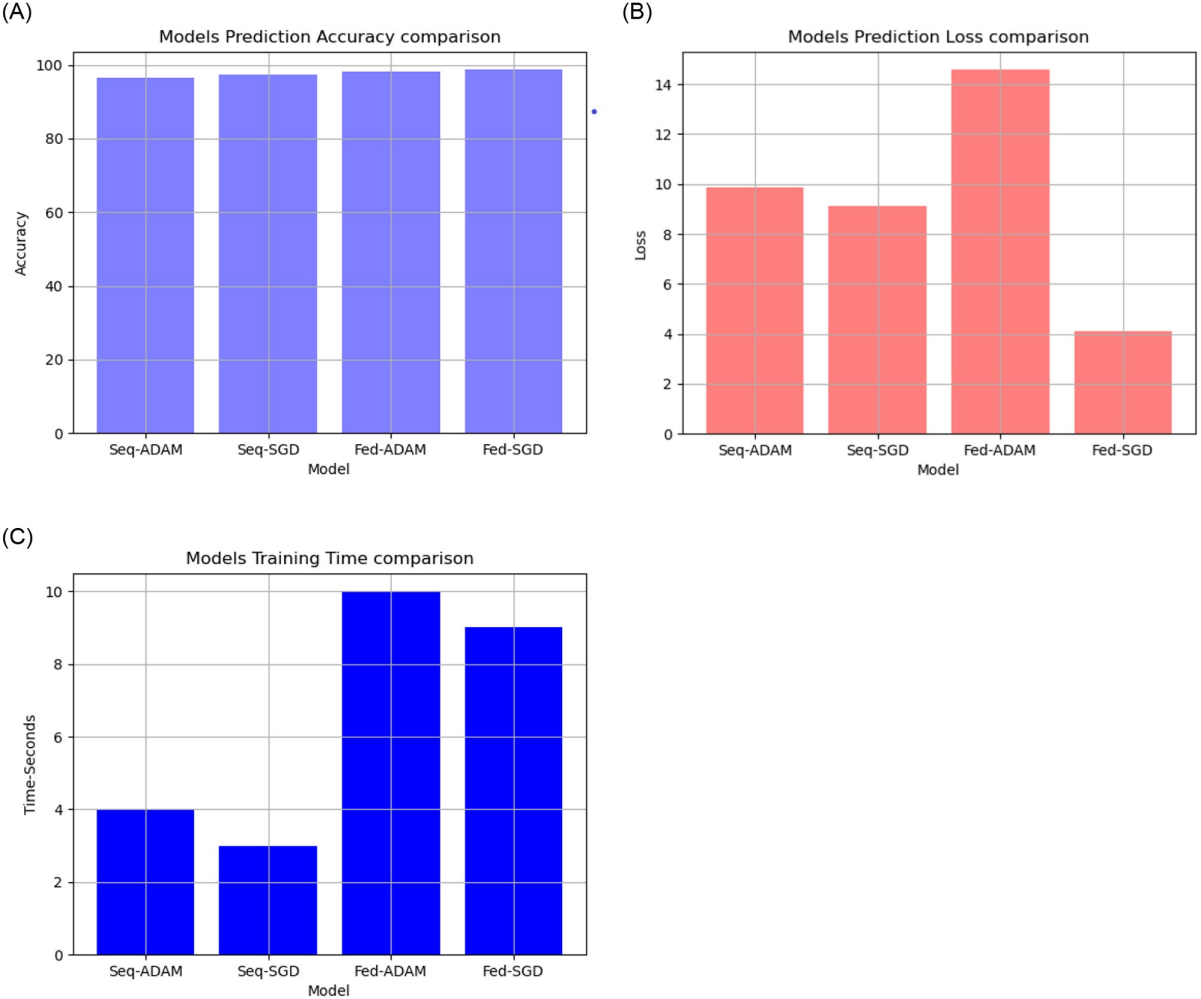
Model accuracy, loss and time comparison on patient’s chest x-rays dataset.

After training the federated and traditional models were used to predict the outcome for a patient (COVID- 19, pneumonia) based on the chest x-ray image. We found that:

- The proposed federated model with SGD algorithm had a higher prediction accuracy than the proposed traditional model As shown in Fig 7 and Table 2.
- The proposed federated model with SGD algorithm had a lower prediction loss than the proposed traditional model As shown in Fig 7 and Table 2.
- The proposed federated model had a high training time than the proposed traditional model As shown in Fig 7 and Table 2.

**Table 2:** Comparison between proposed models accuracy and loss on patient’s chest x-ray datasets.

Criteria/Model	Sequential	Sequential	Federated	Federated
Optimizer	ADAM	SGD	Adam	SGD
learning rate	0.0001	0.0001	0.0001	0.0001
Loss Function	binary crossentropy	binary crossentropy	SparseCategorical Crossentropy	SparseCategorical Crossentropy
Accuracy Function	accuracy	accuracy	SparseCategorical Accuracy	SparseCategorical Accuracy
Model Loss	9.85%	9.13%	14.58%	4.12%
Model Accuracy	96.64%	97.34%	98.28%	98.72%
Training Time	4 minutes	3 minutes	9 minutes	10 minutes

#### Ternary classifier

**Fig 8:**
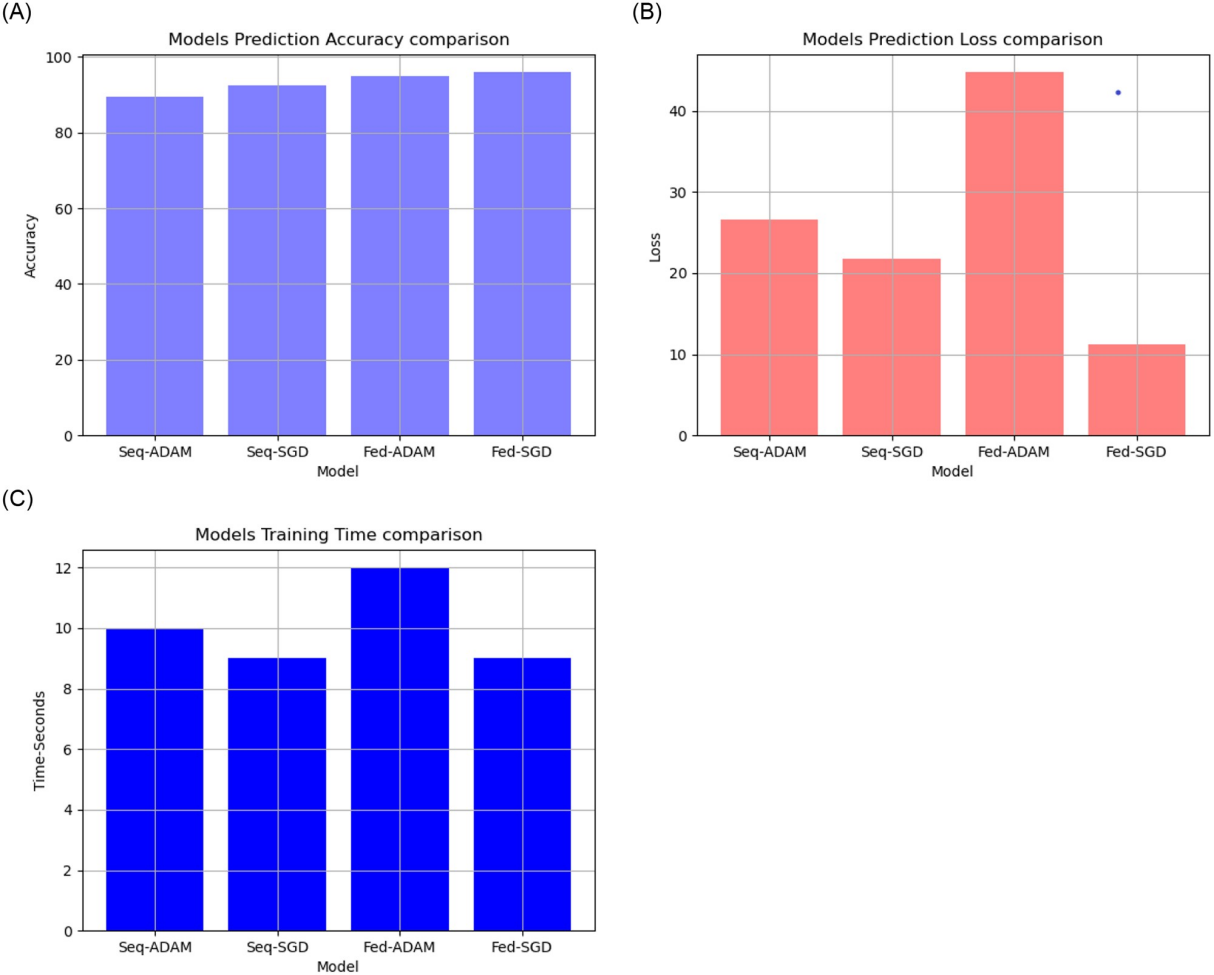
Model accuracy, loss and time comparison on patient’s chest x-rays dataset.

After training, the federated and traditional models were used to predict the patient status (COVID- 19, pneumonia, normal) based on the chest x-ray image. We found that:

- The proposed federated model with SGD algorithm had a higher prediction accuracy than the proposed traditional model As shown in Fig 8 and Table 3.
- The proposed federated model with SGD algorithm had a lower prediction loss than the proposed traditional model as shown in Fig 8 and Table 3.
- The proposed federated model with SGD algorithm had a training time equal or slightly greater than the proposed traditional model as shown in Fig 8 and Table 3.

**Table 3:** Comparison between proposed models accuracy and loss on patient’s chest x-ray.

Criteria/Model	Sequential	Sequential	Federated	Federated
Optimizer	ADAM	SGD	Adam	SGD
learning rate	0.0001	0.0001	0.0001	0.0001
Loss Function	binary crossentropy	binary crossentropy	SparseCategorical crossentropy	SparseCategorical crossentropy
Accuracy Function	accuracy	accuracy	SparseCategorical accuracy	SparseCategorical accuracy
Model Loss	26.65%	21.75%	44.77%	11.24%
Model Accuracy	89.58 %	92.34%	94.82%	95.96%
Training Time	10 minutes	9 minutes	12 minutes	9 minutes

### Hardware specifications

Our experiments were conducted by machine was shown in Table 4

**Table 4:** Hardware specifications for the machine used during about experiments.

Criteria	Specification
CPU	Intel Core i7–6700HQ
GPU	NVIDIA GeForce GTX 950M (4GB DDR3)
STORAGE	256GB SSD + 1000GB HDD
RAM	16GB DDR3L, 2133 MHz

## Discussion

### Datasets

In this work, two types of COVID-19 datasets were used:

- Patients chest x-ray radiography images (CXR) with COVID19, PNEUMONIA, and NORMAL images were obtained from https://www.kaggle.com/prashant268/chest-xray-covid19-pneumonia.
- Patients descriptive datasets with COVID-19 infected cases reported by WHO in Wuhan City, Hubei Province of China from 31 December 2019 provided by https://www.kaggle.com/sudalairajkumar/novel-corona-virus-2019-dataset.
  - Patient’s chest x-ray radiography (CXR) images datasets.
    Dataset contains chest x-ray radiography images (CXR) with COVID19, PNEUMONIA, NORMAL images cases were obtained from www.kaggle.com, the dataset contains 5144 images categorized as follows:
    * 3,418 images for pneumonia cases
    * 1,266 images for normal cases
    * 460 images for COVID-19 cases
  - Patient’s descriptive COVID-19 datasets contained COVID-19 infected cases information (1,085 samples).
    Modifications were required before feeding the machine learning model with the data like:
    * Remove unique columns.
    * Replace null values.
    * Normalize columns like age column instead of (male, female) to be (0, 1).
    * Remove string columns.

The following Table 5: contains the dataset columns description and the action taking with the data to appropriate for machine learning model training.

**Table 5:** Patients descriptive datasets contains COVID-19 infected cases which reported in Wuhan City.

Column Name	Description Description	Data Type	Contains Null	Action
ID	case id unique	number	no	removed
Case Country	(1) yes	number	yes	
	(0) no			
Reporting Date	reporting date	date	yes	
Summary	case summary	string	yes	removed
Location	city inside country	string	yes	
Country	case country	string	yes	
Gender	male, female	string	yes	
Age	age no	number	yes	
symptom onset	start date	date	yes	
If onset approximated	(1) yes	number	yes	
	(0) no			
hosp visit date	visiting date	date	yes	
exposure start	exposure date	date	yes	
exposure end	exposure date end	date	yes	
visiting Wuhan	(1) yes	number	yes	
	(0) no			
from Wuhan	(1) yes	number	yes	
	(0) no			
Death	(1) yes	number	yes	
	(0) no			
recovered	(1) yes	number	yes	
	(0) no			
symptom	synmptom description	string	yes	removed
Source	case registration source	string	yes	removed
Link	case link	string	yes	removed

## Results discussion

**Fig 9:**
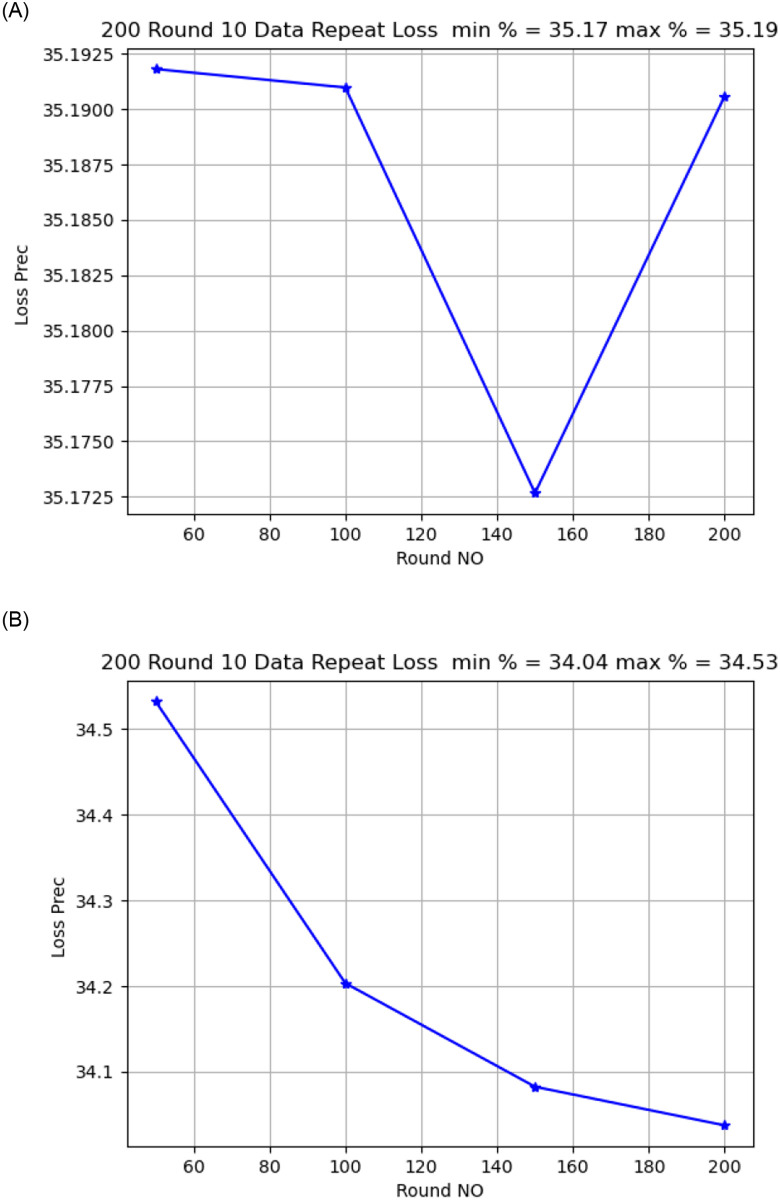
Optimizer loss comparison.

**Fig 10:**
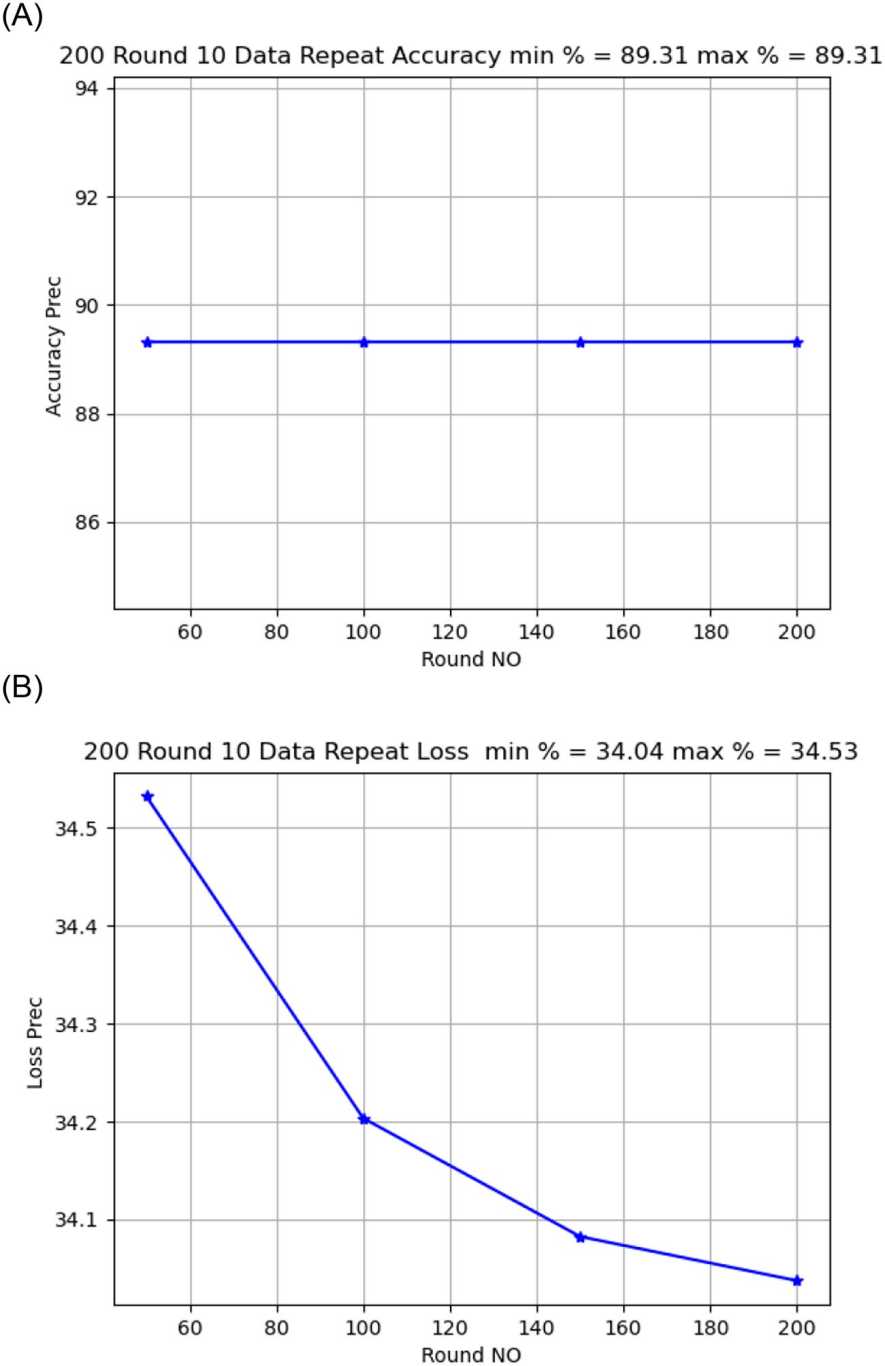
Learning rate comparison.

**Fig 11:**
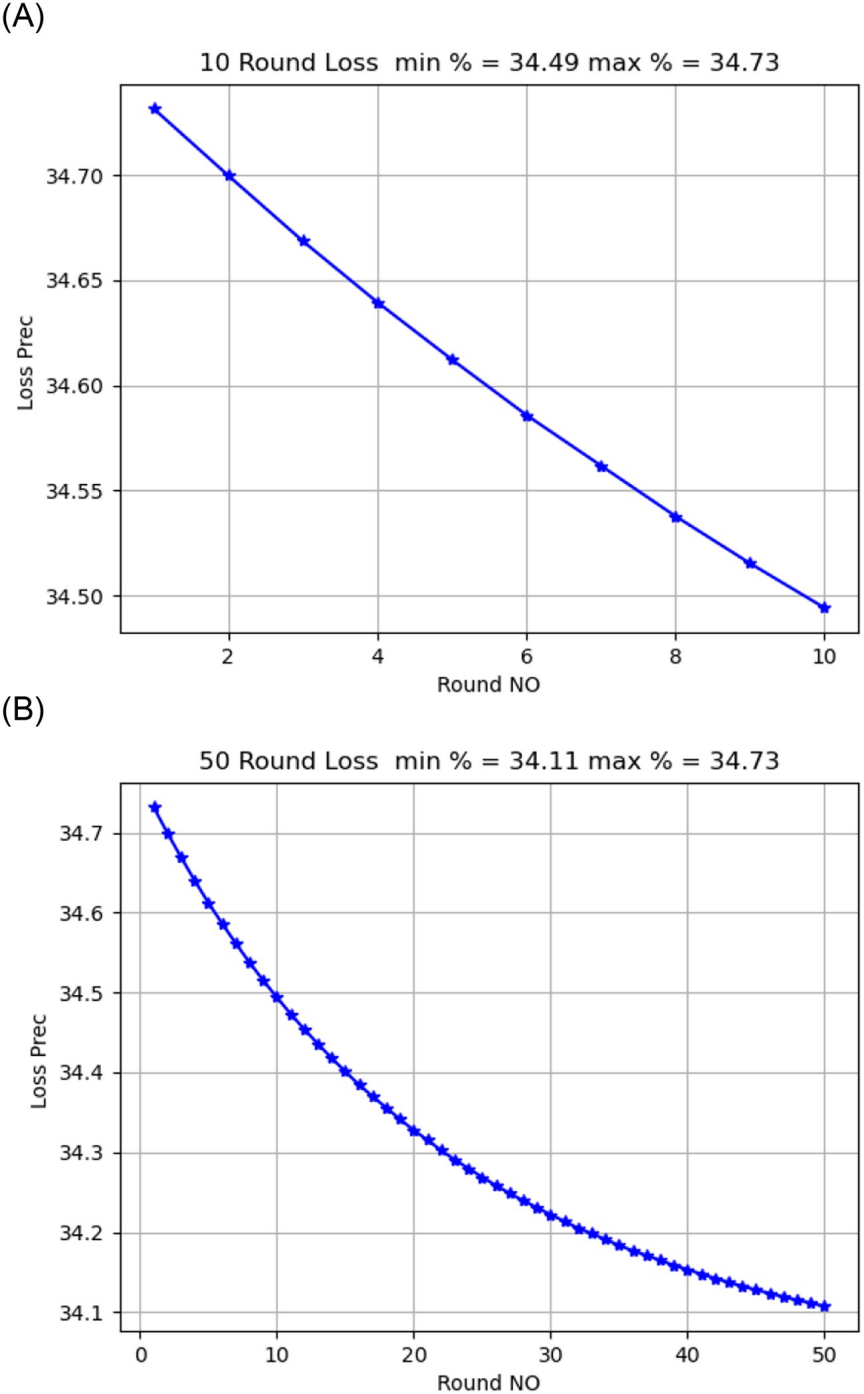
Model loss comparison for 10, 50 round.

**Fig 12:**
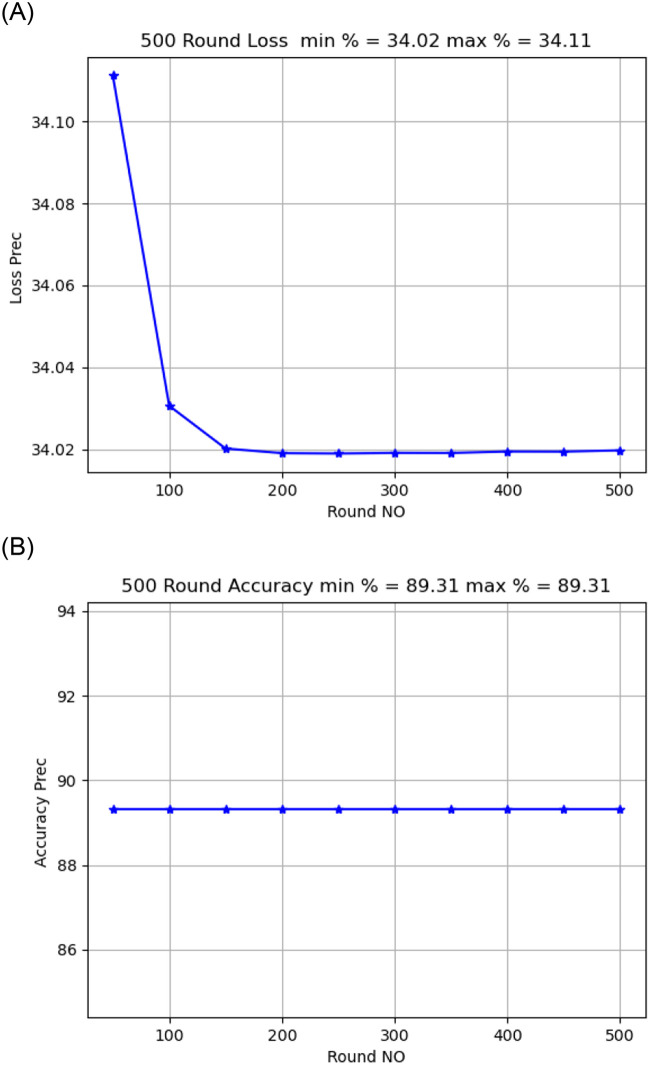
Model accuracy and loss comparison for 500 round.

**Fig 13:**
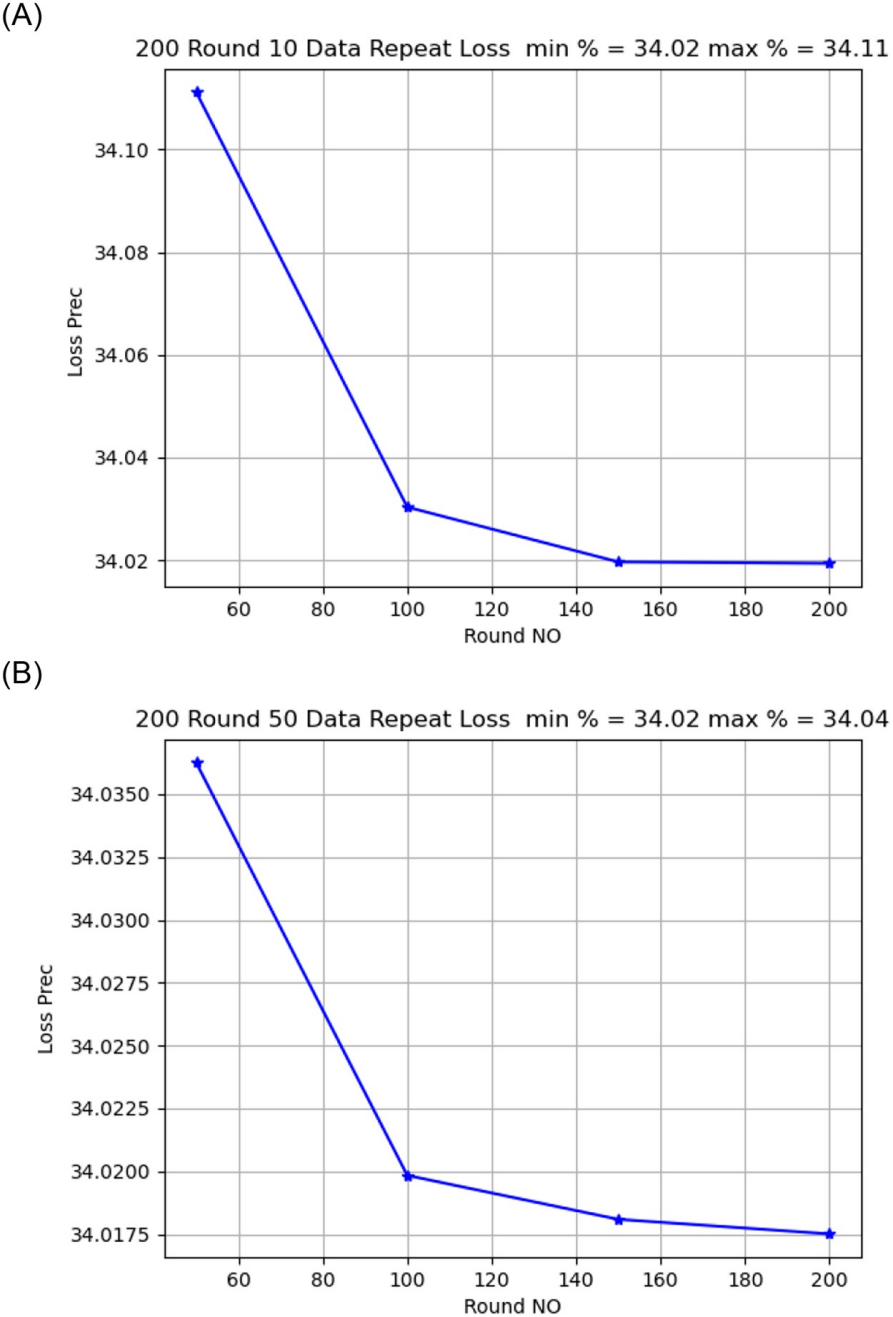
Model loss comparison for 10, 50 round.

**Fig 14:**
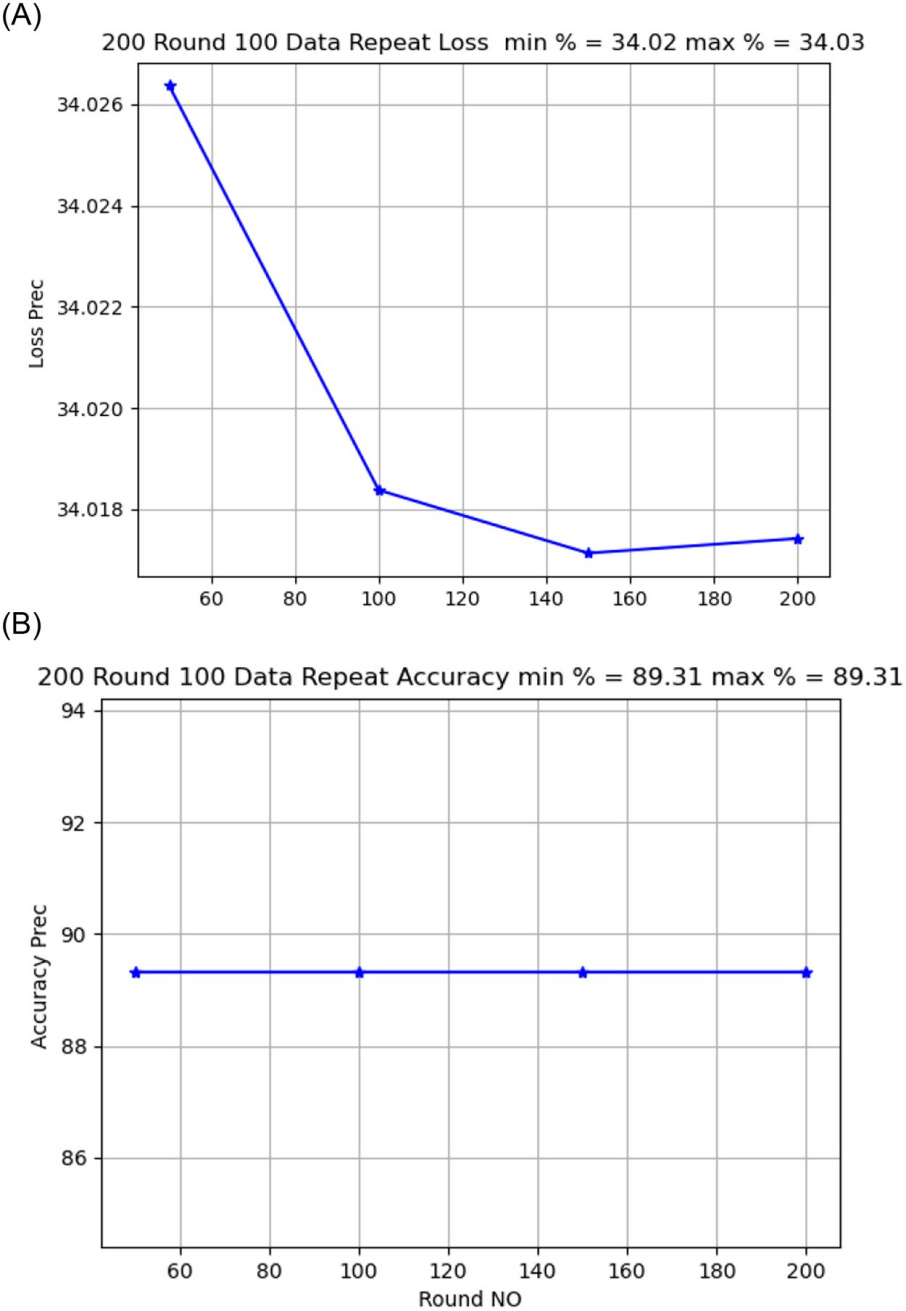
Model accuracy and loss comparison for 100 times data size.

The model parameters modified multiple times to achieve maximum accuracy and minimum loss. These modifications included:

- Activation function
- Model optimizer
- Learning rate
- Number of rounds
- Data Size

1. Activation function
  The sigmoid activation function was more accurate than the relu activation function.
2. Model Optimizer
  Changing the SGD provided a better model accuracy and loss than ADAM, As shown in Fig 9.
3. Learning Rate:
  it is found that changing the learning rate had a slight effect on model accuracy and model loss, when changing the learning rate from 0.02 to 0.01, the model loss changed from 34.02 to 34.04, As shown in Fig 10.
4. Number of rounds:
  Increasing the round number affects the loss but not model accuracy, As shown in Figs 11 and 12.
5. Data Size
  Increasing the data size did not affect the model loss or accuracy, As shown in Figs 13 and 14.

## Conclusion

We applied a proposed federated learning model on COVID-19 datasets, and found that

- The proposed federated learning model gives better prediction accuracy than traditional deep learning model.
- The proposed federated learning model gives a lower loss than traditional machine learning model.
- The proposed federated learning model takes a higher training time than traditional machine learning model.

### The model’s parameters were changed many times to achieve maximum accuracy, minimum loss, and minimum training time, and we found that

- Activation function.
  The softmax activation function was more accurate than relu, sigmoid activation function when applied to the chest x-ray (CXR) images dataset and patient’s descriptive data.
- Model optimizer
  Changing the SGD provided better model accuracy and loss than ADAM when applied on patient’s descriptive data and patient’s chest x-ray (CXR) images dataset.
- Learning rate
  Changing the learning rate had a slight effect on model accuracy and model loss when applied on patient’s descriptive data and chest x-ray (CXR) images dataset.
- Number of rounds
  Increasing the number of rounds had a good effect on reducing the loss but had no impact on model’s accuracy when applied to the patient metadata and the chest x-ray image (CXR) data set.
- Data Size
  Increasing data size did not affect the model loss or model accuracy when applied to patient’s descriptive data and chest x-ray (CXR) images dataset.

**Swarm intelligence algorithms will be used in the future to optimize the proposed federated model for global optimization and reduce the communications overhead. The hybrid model should be tested on chest x-ray radiography (CXR), and chest computed tomography**

## Supporting information

S1 Dataset (RAR)

## References

[1] Liu, Boyi, et al. Experiments of federated learning for covid-19 chest x-ray images.” arXiv preprint arXiv:2007.05592 (2020).

[2] Fiorino Gionata, et al. Inflammatory bowel disease care in the COVID-19 pandemic era: the Humanitas, Milan, experience.” Journal of Crohn’s and Colitis 14.9 (2020): 1330–1333. doi: 10.1093/ecco-jcc/jjaa058 32211765 PMC7184487

[3] Wieczorek Michał, Jakub Siłka, and Marcin Woźniak. Neural network powered COVID-19 spread forecasting model.” Chaos, Solitons Fractals 140 (2020): 110203. doi: 10.1016/j.chaos.2020.110203 32834663 PMC7428770

[4] Zhang Weishan, et al. Dynamic fusion-based federated learning for COVID-19 detection. IEEE Internet of Things Journal (2021). 10.1109/JIOT.2021.3056185 PMC9128757 35663640

[5] Lian, Xiangru, et al. Can decentralized algorithms outperform centralized algorithms? a case study for decentralized parallel stochastic gradient descent. arXiv preprint arXiv:1705.09056 (2017).

[6] Yang Qiang, et al. Federated machine learning: Concept and applications. ACM Transactions on Intelligent Systems and Technology (TIST) 10.2 (2019): 1–19. doi: 10.1145/3298981

[7] Li Tian, et al. Federated learning: Challenges, methods, and future directions IEEE Signal Processing Magazine 37.3 (2020): 50–60. doi: 10.1109/MSP.2020.2975749

[8] Pang, Junjie, et al. Collaborative City Digital Twin For Covid-19 Pandemic: A Federated Learning Solution. arXiv preprint arXiv:2011.02883 (2020).

[9] Afshar Parnian, et al. Covid-caps: A capsule network-based framework for identification of covid-19 cases from x-ray images. Pattern Recognition Letters 138 (2020): 638–643. doi: 10.1016/j.patrec.2020.09.010 32958971 PMC7493761

[10] He, Chaoyang, Murali Annavaram, and Salman Avestimehr. Fednas: Federated deep learning via neural architecture search. arXiv preprint arXiv:2004.08546 (2020).

[11] Ahmad Amir, et al. The number of confirmed cases of covid-19 by using machine learning: Methods and challenges. Archives of Computational Methods in Engineering (2020): 1–9. (2020). doi: 10.1007/s11831-020-09472-8 32837183 PMC7399353

[12] Tsiknakis Nikos, et al. Interpretable artificial intelligence framework for COVID‑19 screening on chest X‑rays. Experimental and Therapeutic Medicine 20.2 (2020): 727–735. doi: 10.3892/etm.2020.8797 32742318 PMC7388253

[13] Otoom Mwaffaq, et al. An IoT-based framework for early identification and monitoring of COVID-19 cases. Biomedical Signal Processing and Control 62 (2020): 102149. arXiv:2008.07343 (2020). doi: 10.1016/j.bspc.2020.102149 32834831 PMC7428786

[14] Nguyen, Thanh Thi. Artificial intelligence in the battle against coronavirus (COVID-19): a survey and future research directions.” arXiv preprint

[15] Salman, Fatima M., et al. Covid-19 detection using artificial intelligence. (2020).

[16] Das, N. Narayan, et al. Automated deep transfer learning-based approach for detection of COVID-19 infection in chest X-rays. Irbm (2020). 10.1016/j.irbm.2020.07.001 PMC7333623 32837679

[17] THaque, A. K. M., et al. Insight about Detection, Prediction and Weather Impact of Coronavirus (COVID-19) using Neural Network. arXiv preprint arXiv:2104.02173 (2021).

[18] Mukherjee Himadri, et al. Deep neural network to detect COVID-19: one architecture for both CT Scans and Chest X-rays. Applied Intelligence (2020): 1–13. 10.1007/s10489-020-01943-6 PMC7646727 34764562

[19] FIBRIANI, Ike, et al. Multi Deep Learning to Diagnose COVID-19 in Lung X-Ray Images with Majority Vote Technique.

[20] Panwar Harsh, et al. Application of deep learning for fast detection of COVID-19 in X-Rays using nCOVnet. Chaos, Solitons Fractals 138 (2020): 109944. doi: 10.1016/j.chaos.2020.109944 32536759 PMC7254021

[21] Vaid, Shashank, Caglar Cakan, and Mohit Bhandari. Using machine learning to estimate unobserved COVID-19 infections in North America. The Journal of bone and joint surgery. American volume (2020). 10.2106/JBJS.20.00715 PMC7396213 32618918

[22] Carrillo-Larco Rodrigo M., and Castillo-Cara Manuel. Using country-level variables to classify countries according to the number of confirmed COVID-19 cases: An unsupervised machine learning approach. Wellcome Open Research 5 (2020). 10.12688/wellcomeopenres.15819.1 PMC7308996 32587900

[23] Khmaissia, Fadoua, et al. An unsupervised machine learning approach to assess the zip code level impact of covid-19 in nyc. arXiv preprint arXiv:2006.08361 (2020).

[24] Khanday Akib Mohi Ud Din, et al. Machine learning based approaches for detecting COVID-19 using clinical text data. International Journal of Information Technology 12.3 (2020): 731–739. doi: 10.1007/s41870-020-00495-9 32838125 PMC7325639

[25] Manavalan R. Automatic identification of diseases in grains crops through computational approaches: A review. Computers and Electronics in Agriculture 178 (2020): 105802. doi: 10.1016/j.compag.2020.105802

[26] Ardabili Sina F., et al. Covid-19 outbreak prediction with machine learning. Algorithms 13.10 (2020): 249. doi: 10.3390/a13100249

[27] Kassani, Sara Hosseinzadeh, et al. Automatic detection of coronavirus disease (covid-19) in x-ray and ct images: A machine learning-based approach. arXiv preprint arXiv:2004.10641 (2020). 10.1016/j.bbe.2021.05.013 PMC8179118 34108787

[28] Iwendi, Celestine, et al. Classification of COVID-19 individuals using adaptive neuro-fuzzy inference system. Multimedia Systems (2021): –-15. 10.1007/s00530-021-00774-w PMC8004563 33814730

[29] Javed Abdul Rehman, et al. A collaborative healthcare framework for shared healthcare plan with ambient intelligence. Human-centric Computing and Information Sciences 10.1 (2020): 1–21. doi: 10.1186/s13673-020-00245-7

[30] Bhattacharya Sweta, et al. Deep learning and medical image processing for coronavirus (COVID-19) pandemic: A survey. Sustainable cities and society 65 (2021): 102589. doi: 10.1016/j.scs.2020.102589 33169099 PMC7642729

[31] Manoj, Mk, et al. An Incentive Based Approach for COVID-19 planning using Blockchain Technology. 2020 IEEE Globecom Workshops (GC Wkshps. IEEE, 2020.

[32] Reddy Thippa G., et al. Hybrid genetic algorithm and a fuzzy logic classifier for heart disease diagnosis. Evolutionary Intelligence 13.2 (2020): 185–196. doi: 10.1007/s12065-019-00327-1

[33] Ulhaq, Anwaar, and Oliver Burmeister. COVID-19 Imaging Data Privacy by Federated Learning Design: A Theoretical Framework. arXiv preprint arXiv:2010.06177 (2020).

[34] Cavoukian Ann. Privacy by design: The 7 foundational principles. Information and privacy commissioner of Ontario, Canada 5 (2009): 12.

